# How do adolescents with short sleep duration spend their extra waking hours? A device-based analysis of physical activity and sedentary behaviour in a Brazilian sample

**DOI:** 10.5935/1984-0063.20200100

**Published:** 2021

**Authors:** Bruno Gonçalves Galdino da Costa, Jean-Philippe Chaput, Marcus Vinicius Veber Lopes, Luís Eduardo Argenta Malheiros, Kelly Samara Silva

**Affiliations:** 1 Universidade Federal de Santa Catarina, Centro de Desportos - Florianópolis - Santa Catarina - Brazil.; 2 Healthy Active Living and Obesity Research Group, Children’s Hospital of Eastern Ontario Research Institute - Ottawa - Ontario - Canada.

**Keywords:** Physical Activity, Sedentary Behaviour, Accelerometer, Sleep Duration, Youth, Public Health

## Abstract

**Objectives:**

To compare sedentary behaviour and physical activity between short sleepers and adequate sleepers in a sample of Brazilian adolescents.

**Material and Methods:**

688 adolescents wore accelerometers on the non-dominant wrist for seven days. Sleep duration, sedentary behaviour, light (LPA), moderate (MPA), and vigorous physical activity (VPA) were estimated. Participants were classified as short (<8h/night) or adequate sleepers (≥8h/night). The minutes and the percentage of time spent in each waking behaviour was compared between short and adequate sleepers.

**Results:**

Participants were 16.3 years old, 50.4% were female, and 67.7% were short sleepers. Adequate sleepers engaged in less (min/day) sedentary behaviour (-53.46), LPA (-25.44), MPA (-4.27), and VPA (-0.63) compared to short sleepers. However, no differences were observed for the proportion of time (68% in sedentary behaviour, 28% in LPA, 3% in MPA, and <0.4% in VPA).

**Conclusion:**

Patterns of waking behaviours are similar between short and adequate sleepers.

## INTRODUCTION

Sleep, sedentary behaviour, and physical activity of different intensities compose the 24-hour cycle of the day, where any minute spent in one behaviour is reduced from the others^1^. Consequently, those who sleep less have more waking time available to engage in sedentary behaviour and/or physical activity. However, research has suggested that due to the effects of sleep deprivation on hormones and sleepiness, the pattern of waking behaviours of short sleepers may not be as active as individuals with sufficient sleep, even if they have more time available^2^.

Sleeping adequately can provide many benefits for health^3,4^, and the lack of it causes drowsiness, daytime sleepiness, and may even impair emotional regulation and cognitive performance^5^. The negative effects of sleep deprivation may predispose children and adolescents who sleep less to opt for sedentary activities instead of physical activity, even if they have more waking time to engage in physical activities. In fact, some studies show an inverse relationship between sleep duration and sedentary behaviour in paediatric samples^2,6^, including an experimental study that showed that a decrease of 1.5 hours in sleep duration increased sedentary behaviour by 0.5 hour/day^7^. However, the effect of short sleep duration on physical activity is not as clear. For example, a study with Spanish children observed that physical activity during the day was associated with shorter sleep duration^8^, whereas findings from a Czech study reported that youth who sleep more were more active compared with youth with short sleep duration^2^. Lastly, it is not clear if the pattern of waking behaviours between short and adequate sleepers is proportionally different, as most studies included comparisons of absolute values (i.e., in minutes per day) and did not report the proportion of waking behaviours (i.e., percentage of the day in sedentary behaviour and physical activity).

Collectively, the relationship between sleep duration and waking behaviours is not entirely clear, as results from previous studies are not consistent and mainly relied on self-reported data. In addition, the available evidence focuses on children and young adolescents; less evidence is available for older adolescents^2^ who experience important physiological and behavioural changes that can impact their sleep and activity behaviours during this period^9,10^. Lastly, the available evidence greatly relies on studies conducted in higher-income countries, and less is known in middle-income settings. Thus, the aim of the present study was to compare sedentary behaviour and physical activity profiles between short sleepers and adequate sleepers in a sample of Brazilian adolescents.

## MATERIAL AND METHODS

### Study sample

**Figure 1:**
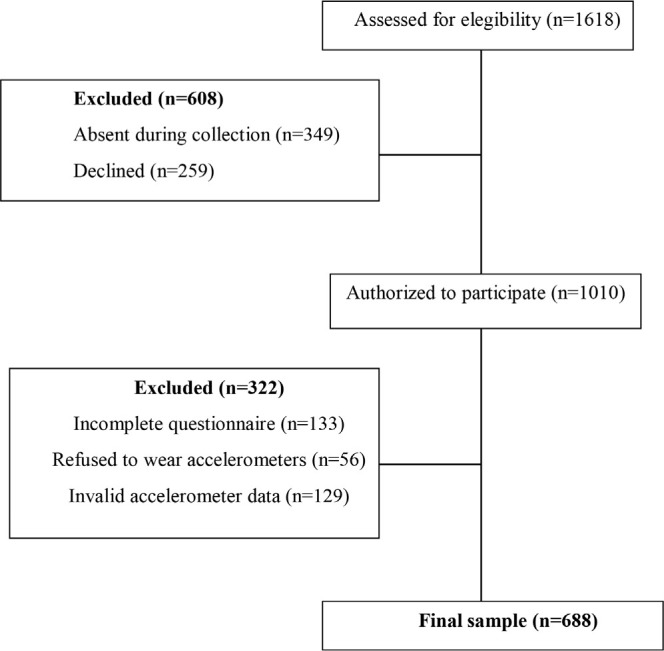
Flowchart of participants included in the study.

Participants were recruited from all three schools that offered high school integrated with professional courses in the mesoregion Greater Florianópolis, Southern Brazil. A total of 1,618 students were enrolled in these schools, and all eligible high school students who were present during data collection, between August and December 2019, were invited to participate (n=1249). Consent forms were obtained from the students and from their legal guardians (n=1010), and 688 participants provided valid measurements on all study variables and were included in the present analyses. More details regarding the exclusions can be observed on Figure 1. The research project was approved by the ethics committee in research with human beings of the Universidade Federal de Santa Catarina (protocol number: 3.168.745).

### Measures

Actigraph GT3X+ and wGT3X+ accelerometers (ActiGraph Corp., Pensacola, FL, USA) were used to measure sleep duration, sedentary behaviour, and physical activity of light, moderate, and vigorous intensity. Participants were given the devices during class time, and were oriented to wear it for seven days, 24 hours per day, taking it off only if the accelerometer would be submerged in water (e.g., surfing, swimming, but not for showering or washing dishes). The accelerometers were positioned on the non-dominant wrist, secured by a disposable PVC band. Participants who provided 16 hours of valid data in four or more days, including a weekend day, were included in the analyses (n=688). Acceleration data were imputed on non-wear time periods using data from the days with valid wear-time. More information about the imputation process can be found elsewhere^11^. Acceleration was classified into activity intensities using the cut-off points proposed by Hildebrand et al. (2014)^12^ and Hildebrand et al. (2017)^13^. Sleep duration was estimated using the Heuristic algorithm looking at Distribution of Change in Z-Angle^14^.

A measurement day was considered as the time interval between wake-up times (e.g., one day may begin at 8 a.m., and end at 9 a.m. the next day), meaning that a day cycle could have more or less than 24 total hours. All behaviours were then averaged between data collection days (4-7 days), and weighted to 1,440 minutes. Sleep duration was dichotomized into short sleepers (<8 hours/night) and adequate sleepers (≥8 hours/night) based on sleep duration recommendations^15^. Accelerometers were initialized and data were downloaded using the Actilife software, version 6.8.11., for Windows, and analyses of raw accelerometer data were conducted using the GGIR package^11^.

### Statistical analysis

Participants’ characteristics were described using means and standard deviations, and relative and absolute frequencies for continuous and categorical variables, respectively. Differences in sedentary behaviour and in light physical activity between short and adequate sleepers were tested using multilevel linear regression analyses. For moderate and vigorous physical activity, generalized linear multilevel models were used with the Gamma family. For each waking behaviour, a model was fit for minutes/day, and another model was fit using the proportion of waking time spent on that behaviour relative to the sum of the others. Models were adjusted for sex, age, and maternal educational level, and a random intercept for the schools was estimated. Analyses were conducted with R (R Foundation for Statistical Computing, Vienna, Austria), version 3.6.0 for Windows, using the lme4 package. Significance was set at *p*<0.05 (two-tailed).

## RESULTS

Characteristics of the study sample (n=688) are shown in Table 1. The sociodemographic characteristics of the sample were compared between short and adequate sleepers. The proportion of males among short sleepers (54.1%) was higher than those in the adequate sleeper’s group (40.1%; *p*<0.05). No statistically significant group differences were observed for age or mother’s education.

**Table 1:** Characteristics of the sample (mean ± SD or n (%).

	Mean/n	±SD/(%)
**Sex**		
Male	341	(49.6)
Female	347	(50.4)
**Age**	16.34	±1.08
**Mother's education (years)**		
<8	67	(9.7)
8-11 years	244	(35.5)
>11	354	(51.5)
Unknown	23	(3.3)
**Sleep**		
<8 hours/night	466	(67.7)
≥8 hours/night	222	(32.3)
**Waking behaviours (minutes/day)**		
Sedentary behaviour	616.87	±81.14
Light physical activity	252.87	±58.73
Moderate physical activity	29.13	±15.05
Vigorous physical activity	2.57	±6.21

SD: Standard deviation.

Differences in minutes of sedentary behaviour and physical activity indicators between short and adequate sleepers can be observed in Table 2. Significant differences were found when absolute minutes per day were observed, with short sleepers engaging in a higher volume of all waking behaviours compared to adequate sleepers. However, when proportion of wake-up time spent on each behaviour was analysed, no significant differences were observed.

**Table 2:** Differences in the waking-time behaviours between short (<8 h/night) and adequate (≥8 h/night) sleepers (n=688).

	Short sleepers Mean (SD)	Adequate sleepers Mean (SD)	Difference (95% CI)
Minutes/day			
Sedentary behaviour (minutes/day)^a^	673.23 (76.79)	618.96 (65.78)	**-53.46 (-64.87;-41.92)**
Light physical activity (minutes/day)^a^	276.83 (62.47)	252.28 (56.92)	**-25.44 (-34.98;-16.03)**
Moderate physical activity (minutes/day)^b^	32.59 (16.26)	27.57 (14.44)	**-4.27 (-6.56;-1.97)**
Vigorous physical activity (minutes/day)^b^	3.08 (7.97)	2.02 (2.73)	**-0.63 (-1.25;-0.02)**
Sleep duration (minutes/night)^a^	424.18 (40.05)	513.18 (26.57)	**83.92 (77.80; 90.33)**
Proportion of waking behaviours			
Sedentary behaviours (%)^a^	68.30 (7.17)	68.67 (6.95)	0.41 (-0.7; 1.52)
Light physical activity (%)^a^	28.08 (6.19)	28.04 (6.23)	-0.21 (-1.17; 0.75)
Moderate physical activity (%)^b^	3.31 (1.65)	3.06 (1.60)	-0.18 (-0.42; 0.07)
Vigorous physical activity (%)^b^	0.31 (0.78)	0.22 (0.30)	-0.05 (-0.11; 0.01)

SD: Standard deviation; 95% CI: 95% confidence intervals; a: Multilevel linear regressions; b: Generalized linear multilevel models with Gamma family; Bold values indicate statistical significance at p<0.05.

## DISCUSSION

This article aimed at comparing physical activity and sedentary behaviours between short and adequate sleepers in a sample of Brazilian adolescents. Our results suggest that although significant differences are observed for the time engaged in sedentary behaviours and physical activities (min/day), adequate sleepers engage in the same relative amount of behaviours (%) during their waking time compared to short sleepers. This finding contrasts with previous studies^2,6,16^, and may indicate that Brazilian high school adolescents may have a different behavioural pattern during the day compared to children and adolescents in other settings. This indicates that although absolute differences exist because of time displacement, both in terms of sedentary behaviour and physical activity, the proportion of behaviours is similar with no preference for sedentary time over physical activity with less sleep. However, previous studies did not compare the proportion of waking behaviours, so it was not clear if the pattern of behaviours during the day also changed proportionally in a similar manner in these studies.

A study with Mexican American children and adolescents has suggested that increasing sleep duration may be an effective strategy to reduce sedentary behaviour^16^, which should have positive impacts on health. Other studies also support that longer sleep duration is associated with more physical activity and lower sedentary behaviour^2,6^. However, our results suggest that longer sleep duration is associated with less physical activity and with less sedentary behaviour. These results suggest a conundrum, where it is not clear if the increased time in sleep duration and decreased time in sedentary behaviour would be better for health at the expense of less physical activity. Given that increasing physical activity^17^, getting adequate levels of sleep^4^, and reducing sedentary behaviours^18^ are all good for the health of adolescents, interventions and policies would have to target both short and adequate sleepers in changing their waking behaviours patterns.

This study has limitations, such as the cross-sectional design that precludes establishing causal inference and the limited generalizability of the findings to the population studied. Another limitation is a possible loss of accuracy of the behaviour estimates for participants who have removed the accelerometer for water-based activities. However, this study also has strengths, such as the integrated measurement of device-based 24-hour data, and the inclusion of a sample of adolescents in a middle-income country, which is not very common in the scientific literature.

## CONCLUSION

In conclusion, short sleepers engaged in more absolute sedentary behaviour and physical activities compared to adequate sleepers, but they engaged in proportionally the same amount of waking behaviours (~68% of the time sedentary, 28% in light physical activity, 3% in moderate physical activity, and less than 1% in vigorous activities). Future studies should prospectively analyse if this pattern is kept during the transition to adulthood and how it affects health. Experimental studies should also aim to investigate the effects of changing sleep or waking behaviours on each other and examine the impact on various health outcomes.
